# A Pocket Text-Book of Dermatology

**Published:** 1903-01

**Authors:** 


A Pocket Text-Book of Dermatology.—By Joseph Grindon, M. D., Professor of Clinical Dermatology and Syphilis in the Medical Department of Washington University, St. Louis. In one 12mo. volume of 367 pages, with 39 illustrations, in black and colors. Lea's Series of Pocket Text-Books. Edited by Bern B. Gallaudet, M. D. Cloth, $2.00, net; limp leather, $2.50, net. Lea Brothers & Co., Publishers, Philadelphia and New York.

Like the other volumes of Lea's Series of Pocket Text-Books, this manual is a comprehensive and trustworthy treatise sufficiently brief to be of use to the student and yet it contains all of the details of etiology, pathology, diagnosis and treatment that the practitioner is likely to require for ready reference.

The author possesses an enviable faculty for expressing himself in the clearest and happiest possible manner in the fewest possible words. R.

